# 2-Hydroxy­imino-1-phenyl­ethanone thio­semicarbazone monohydrate

**DOI:** 10.1107/S1600536808004947

**Published:** 2008-02-22

**Authors:** Nursabah Sarıkavaklı, İknur Babahan, Ertan Şahin, Tuncer Hökelek

**Affiliations:** a Adnan Menderes University, Department of Chemistry, 09010 Aydın, Turkey; b Atatürk University, Department of Chemistry, 22240 Erzurum, Turkey; c Hacettepe University, Department of Physics, 06800 Beytepe, Ankara, Turkey

## Abstract

In the title thio­semicarbazone derivative, C_9_H_10_N_4_OS·H_2_O, intra­molecular N—H⋯N hydrogen bonds result in the formation of two nearly coplanar five- and six-membered rings, which are also almost coplanar with the adjacent phenyl ring. The oxime group has an *E* configuration and is involved in inter­molecular O—H⋯O hydrogen bonding as a donor. In the crystal structure, intra­molecular O—H⋯S and N—H⋯N and inter­molecular O—H⋯O and N—H⋯S hydrogen bonds generate edge-fused *R*_2_^2^(8) and *R*_4_^1^(11) ring motifs. The hydrogen-bonded motifs are linked to each other to form a three-dimensional supra­molecular network.

## Related literature

For general backgroud, see: Lukevics *et al.* (1995 ▶); Liberta & West (1992 ▶); Hagenbach & Gysin (1952 ▶); Jones *et al.* (1965 ▶); Brockman & Thomson (1956 ▶); Klayman *et al.* (1979 ▶); Petering & van Giesen (1966 ▶); Sevagapandian *et al.* (2000 ▶); Forman (1964 ▶); Holan *et al.* (1984 ▶); Balsamo *et al.* (1990 ▶); Marsman *et al.* (1999 ▶); Karle *et al.* (1996 ▶); Etter *et al.* (1990 ▶); Chertanova *et al.* (1994 ▶); Bernstein *et al.* (1995 ▶). For related structures, see: Sarıkavaklı *et al.* (2007 ▶); Özel Güven *et al.* (2007 ▶); Hökelek, Batı *et al.* (2001 ▶); Hökelek, Zülfikaroğlu & Batı (2001 ▶); Büyükgüngör *et al.* (2003 ▶); Hökelek *et al.* (2004*a* ▶,*b* ▶); Hökelek *et al.* (2004 ▶). For the synthesis, see: El-Shazly *et al.* (2005 ▶). For bond-length data, see: Allen *et al.* (1987 ▶).
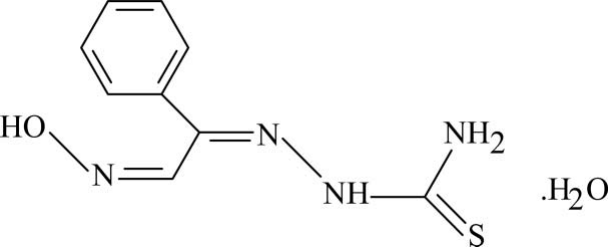


## Experimental

### 

#### Crystal data

C_9_H_10_N_4_OS·H_2_O
*M* *_r_* = 240.29
Monoclinic, 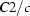

*a* = 28.5615 (3) Å
*b* = 4.6805 (3) Å
*c* = 22.0977 (4) Å
β = 127.24 (2)°
*V* = 2351.8 (6) Å^3^
*Z* = 8
Mo *K*α radiation
μ = 0.27 mm^−1^
*T* = 294 (2) K
0.30 × 0.20 × 0.15 mm

#### Data collection

Rigaku R-AXIS RAPID-S diffractometer
Absorption correction: multi-scan (Blessing, 1995 ▶) *T*_min_ = 0.940, *T*_max_ = 0.960
31269 measured reflections
3607 independent reflections
2146 reflections with *I* > 2σ(*I*)
*R*_int_ = 0.090

#### Refinement

*R*[*F* ^2^ > 2σ(*F* ^2^)] = 0.062
*wR*(*F* ^2^) = 0.154
*S* = 1.04
3607 reflections
193 parameters
8 restraints
H atoms treated by a mixture of independent and constrained refinement
Δρ_max_ = 0.14 e Å^−3^
Δρ_min_ = −0.31 e Å^−3^

### 

Data collection: *CrystalClear* (Rigaku/MSC, 2005 ▶); cell refinement: *CrystalClear*; data reduction: *CrystalClear*; program(s) used to solve structure: *SHELXS97* (Sheldrick, 2008 ▶); program(s) used to refine structure: *SHELXL97* (Sheldrick, 2008 ▶); molecular graphics: *ORTEP-3 for Windows* (Farrugia, 1997 ▶) and *PLATON* (Spek, 2003 ▶); software used to prepare material for publication: *WinGX* (Farrugia, 1999 ▶).

## Supplementary Material

Crystal structure: contains datablocks I, global. DOI: 10.1107/S1600536808004947/xu2403sup1.cif

Structure factors: contains datablocks I. DOI: 10.1107/S1600536808004947/xu2403Isup2.hkl

Additional supplementary materials:  crystallographic information; 3D view; checkCIF report

## Figures and Tables

**Table 1:** Hydrogen-bond geometry (Å, °)

*D*—H⋯*A*	*D*—H	H⋯*A*	*D*⋯*A*	*D*—H⋯*A*
O1—H1*A*⋯O2^i^	0.90 (3)	1.83 (3)	2.728 (3)	174 (3)
O2—H21⋯S1	0.94 (3)	2.32 (3)	3.250 (3)	171 (3)
O2—H22⋯O2^i^	0.91 (3)	1.98 (3)	2.886 (3)	172 (4)
N3—H3*A*⋯N1	0.92 (3)	1.91 (2)	2.604 (3)	130 (2)
N4—H41⋯S1^ii^	0.92 (2)	2.53 (2)	3.434 (2)	169 (3)
N4—H42⋯N2	0.93 (3)	2.24 (3)	2.643 (3)	105 (2)

Symmetry codes: (i) 
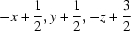
; (ii) 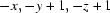.

**Fig. 1.**
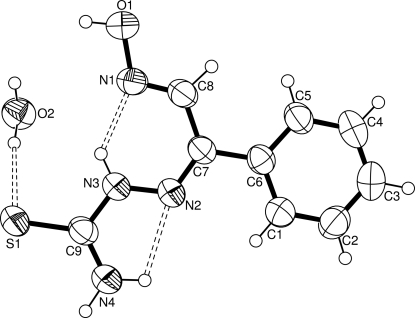
The molecular structure of the title molecule with the atom-numbering scheme. Displacement ellipsoids are drawn at the 50% probability level.

**Fig. 2.**
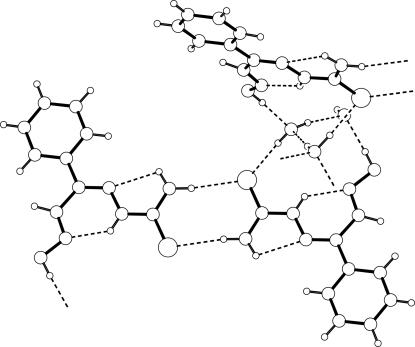
A part of the crystal structure of (I), showing the formation of R22(8) and R41(11) ring motifs. Hydrogen bonds are shown as dashed lines.

**Fig. 3.**
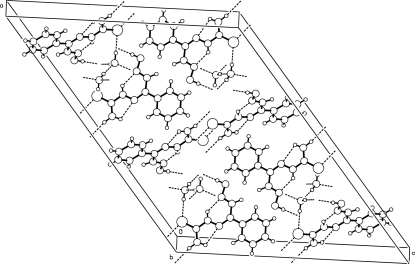
A partial packing diagram of (I). Hydrogen bonds are shown as dashed lines.

## supplementary crystallographic information

### Comment

Thiosemicarbazones are derivatives of carbonyl compounds and they have a wide range of biological activities, depending on the parent aldehyde or ketone (Lukevics *et al.*, 1995; Liberta & West, 1992). Some of the thiosemicarbazone derivatives have antitumour (Hagenbach & Gysin, 1952), antiviral (Jones *et al.*, 1965), antileukaemic (Brockman & Thomson, 1956) and antimalarial (Klayman *et al.*, 1979) activities. Thus, some of them have been used as drugs and have the ability to form complexes (Petering & van Giesen, 1966).

Oxime and dioxime derivatives are very important compounds in the chemical industry and medicine (Sevagapandian *et al.*, 2000). They have a broad pharmacological activity spectrum, encompassing antibacterial, antidepressant and antifungal activities (Forman, 1964; Holan *et al.*, 1984; Balsamo *et al.*, 1990). The oxime (–C=N—OH) moiety is potentially ambidentate, with possibilities of coordination through nitrogen and/or oxygen atoms. It is a functional group that has not been extensively explored in crystal engineering. In the solid state, oximes are usually associated *via* O—H···N hydrogen bonds of length 2.8 Å.

Oxime groups possess stronger hydrogen-bonding capabilities than alcohols, phenols, and carboxylic acids (Marsman *et al.*, 1999), in which intermolecular hydrogen bonding combines moderate strength and directionality (Karle *et al.*, 1996) in linking molecules to form supramolecular structures; this has received considerable attention with respect to directional noncovalent intermolecular interactions (Etter *et al.*, 1990).

The structures of some oxime and dioxime derivatives have been determined in our laboratory, including those of 2,3-dimethylquinoxaline-dimethyl-glyoxime (1/1), [(II) Hökelek, Batı *et al.*, 2001], 1-(2,6-dimethylphenylamino)- propane-1,2-dione dioxime, [(III) (Hökelek, Zülfikaroğlu & Batı, 2001), *N*-hydroxy-2-oxo-2,*N*'-diphenylacetamidine, [(IV) (Büyükgüngör *et al.*, 2003], *N*-(3,4-dichlorophenyl)-*N*'-hydroxy-2-oxo-2-phenylacetamidine, [(V) Hökelek *et al.*, 2004], *N*-hydroxy-*N*'-(1-naphthyl)-2-phenylacetamidin-2-one [(VI) Hökelek *et al.*, 2004*a*], *N*-(3-chloro-4-methylphenyl)-*N*'-hydroxy-2 -oxo-2-phenylacetamidine [(VII) Hökelek *et al.*, 2004*b*], 2-(1*H*-benzimidazol -1-yl)-1-phenylethanone oxime [(VIII) Özel Güven *et al.*, 2007] and (1*Z*,2E)-1-(3,5-dimethyl-1*H*-pyrazole-1-yl)ethane-1,2-dione dioxime [(IX) Sarıkavaklı *et al.*, 2007]. The structure determination of the title compound, (I), a thiosemicarbazone derivative with one 2-hydroxyimino -1-phenyl-ethanone, one thiosemicarbazone moieties and one uncoordinated water molecule, was carried out in order to investigate the strength of the hydrogen bonding capability of the oxime and thiosemicarbazone groups and to compare the geometry of the oxime moiety with the previously reported ones.

In the molecule of the title compound, (I), (Fig. 1) the bond lengths (Allen *et al.*, 1987) and angles are generally within normal ranges. Ring A (C1—C6) is, of course, planar. The intramolecular N—H···N hydrogen bonds (Table 1) result in the formation of two more planar five- and six-membered rings B (C9/N2—N4/H42) and C (C7/C8/N1—N3/H3A). The rings A, B and C are also nearly coplanar with dihedral angles of A/B = 3.47 (10)°, A/C = 2.84 (10) and B/C = 5.94 (10)°.

Some significant changes in the geometry of the oxime moiety are evident when the bond lengths and angles are compared with the corresponding values in compounds (II)-(VII) (Table 2). The oxime moiety has an E configuration [C7—C8—N1—O1 177.1 (2)°; Chertanova *et al.*, 1994]. In this configuration, the oxime groups are involved as donors in O—H···O intermolecular hydrogen bondings (Table 1).

In the crystal structure, intramolecular O—H···S and N—H···N and intermolecular O—H···O and N—H···S hydrogen bonds (Table 1) generate edge-fused *R*_2_^2^(8) and *R*_4_^1^(11) ring motifs (Fig. 2) (Bernstein *et al.*, 1995). The hydrogen bonded motifs are linked to each other to form a three dimensional network (Fig. 3). The intra- and intermolecular hydrogen bonds seem to be effective in the stabilization of the crystal structure.

### Experimental

The title compound was prepared according to the literature method (El-Shazly *et al.*, 2005). 2-Isonitrosoacetophenone (149 mg, 1 mmol) was reacted with thiosemicarbazide (91 mg, 1 mmol) in ethanol-water mixture (1:1) by refluxing for 24 h. Then, a few drops of glacial acetic acid were added. The formed precipitate was filtered and recrystallized from ethanol to obtain yellow crystals (yield: 155 mg, 70%).

### Refinement

H atoms were located in difference syntheses and refined isotropically [O—H = 0.903 (18)–0.940 (17) Å; *U*_iso_(H) = 0.100 (10)–0.133 (14) Å^2^, N—H = 0.915 (17)–0.930 (17) Å; *U*_iso_(H) = 0.077 (8)–0.084 (9) Å^2^ and C—H = 0.92 (3)–0.97 (3) Å; *U*_iso_(H) = 0.071 (8)–0.092 (9) Å^2^]. The restrains on the O—H (for OH) and N—H (for NH and NH_2_) bonds and O—H bond lengths and H—O—H bond angle of water molecule were applied.

### Crystal data

**Table:**

C_9_H_10_N_4_OS·H_2_O	*F*(000) = 1008
*M**_r_* = 240.29	*D*_x_ = 1.357 Mg m^−^^3^
Monoclinic, *C*2/*c*	Melting point: 443 K
Hall symbol: -C 2yc	Mo *K*α radiation, λ = 0.71073 Å
*a* = 28.5615 (3) Å	Cell parameters from 2893 reflections
*b* = 4.6805 (3) Å	θ = 2.3–30.5°
*c* = 22.0977 (4) Å	µ = 0.27 mm^−^^1^
β = 127.24 (2)°	*T* = 294 K
*V* = 2351.8 (6) Å^3^	Prism, yellow
*Z* = 8	0.30 × 0.20 × 0.15 mm

### Data collection

**Table:**

Rigaku R-AXIS RAPID-S diffractometer	3607 independent reflections
Radiation source: fine-focus sealed tube	2146 reflections with *I* > 2σ(*I*)
graphite	*R*_int_ = 0.090
ω scans	θ_max_ = 30.5°, θ_min_ = 2.3°
Absorption correction: multi-scan (Blessing, 1995)	*h* = −40→40
*T*_min_ = 0.940, *T*_max_ = 0.960	*k* = −6→5
31269 measured reflections	*l* = −31→31

### Refinement

**Table:**

Refinement on *F*^2^	Primary atom site location: structure-invariant direct methods
Least-squares matrix: full	Secondary atom site location: difference Fourier map
*R*[*F*^2^ > 2σ(*F*^2^)] = 0.062	Hydrogen site location: inferred from neighbouring sites
*wR*(*F*^2^) = 0.154	H atoms treated by a mixture of independent and constrained refinement
*S* = 1.04	*w* = 1/[σ^2^(*F*_o_^2^) + (0.0474*P*)^2^ + 0.9027*P*] where *P* = (*F*_o_^2^ + 2*F*_c_^2^)/3
3607 reflections	(Δ/σ)_max_ < 0.001
193 parameters	Δρ_max_ = 0.14 e Å^−^^3^
8 restraints	Δρ_min_ = −0.31 e Å^−^^3^

### Special details

**Table:**

Geometry. All e.s.d.'s (except the e.s.d. in the dihedral angle between two l.s. planes) are estimated using the full covariance matrix. The cell e.s.d.'s are taken into account individually in the estimation of e.s.d.'s in distances, angles and torsion angles; correlations between e.s.d.'s in cell parameters are only used when they are defined by crystal symmetry. An approximate (isotropic) treatment of cell e.s.d.'s is used for estimating e.s.d.'s involving l.s. planes.
Refinement. Refinement of *F*^2^ against ALL reflections. The weighted *R*-factor *wR* and goodness of fit *S* are based on *F*^2^, conventional *R*-factors *R* are based on *F*, with *F* set to zero for negative *F*^2^. The threshold expression of *F*^2^ > σ(*F*^2^) is used only for calculating *R*-factors(gt) *etc*. and is not relevant to the choice of reflections for refinement. *R*-factors based on *F*^2^ are statistically about twice as large as those based on *F*, and *R*- factors based on ALL data will be even larger.

### Fractional atomic coordinates and isotropic or equivalent isotropic displacement parameters (Å^2^)

**Table:**

	*x*	*y*	*z*	*U*_iso_*/*U*_eq_	
S1	0.08587 (3)	0.69943 (16)	0.58554 (3)	0.0693 (2)	
O1	0.24428 (8)	0.9359 (4)	0.88734 (10)	0.0740 (5)	
H1A	0.2527 (12)	1.022 (6)	0.8584 (15)	0.100 (10)*	
O2	0.22848 (8)	0.6577 (4)	0.70204 (11)	0.0720 (5)	
H21	0.1878 (8)	0.692 (7)	0.6673 (16)	0.133 (14)*	
H22	0.2445 (13)	0.817 (5)	0.7313 (16)	0.113 (12)*	
N1	0.19953 (8)	0.7460 (4)	0.83853 (10)	0.0576 (5)	
N2	0.09978 (8)	0.3607 (4)	0.75639 (10)	0.0577 (5)	
N3	0.10929 (9)	0.5206 (5)	0.71329 (10)	0.0619 (5)	
H3A	0.1412 (9)	0.641 (5)	0.7347 (14)	0.077 (8)*	
N4	0.02448 (9)	0.3422 (5)	0.60650 (11)	0.0654 (6)	
H41	−0.0011 (10)	0.316 (5)	0.5550 (10)	0.076 (8)*	
H42	0.0210 (12)	0.238 (5)	0.6394 (14)	0.084 (9)*	
C1	0.07415 (11)	0.0295 (6)	0.83508 (14)	0.0644 (6)	
H1	0.0517 (11)	0.012 (5)	0.7826 (15)	0.074 (8)*	
C2	0.06113 (12)	−0.1405 (6)	0.87378 (17)	0.0721 (7)	
H2	0.0280 (11)	−0.257 (5)	0.8455 (15)	0.071 (8)*	
C3	0.09577 (13)	−0.1362 (6)	0.95201 (17)	0.0724 (7)	
H3	0.0859 (12)	−0.245 (6)	0.9780 (16)	0.083 (9)*	
C4	0.14393 (14)	0.0388 (6)	0.99041 (16)	0.0742 (7)	
H4	0.1666 (12)	0.036 (6)	1.0426 (16)	0.092 (9)*	
C5	0.15737 (13)	0.2112 (6)	0.95211 (14)	0.0639 (6)	
H5	0.1913 (13)	0.330 (6)	0.9810 (17)	0.089 (9)*	
C6	0.12248 (9)	0.2111 (5)	0.87338 (12)	0.0521 (5)	
C7	0.13487 (9)	0.3927 (5)	0.82959 (11)	0.0516 (5)	
C8	0.18351 (10)	0.5985 (5)	0.87101 (13)	0.0580 (6)	
H8	0.2005 (11)	0.621 (5)	0.9241 (15)	0.080 (8)*	
C9	0.07134 (10)	0.5057 (5)	0.63645 (11)	0.0556 (5)	

### Atomic displacement parameters (Å^2^)

**Table:**

	*U*^11^	*U*^22^	*U*^33^	*U*^12^	*U*^13^	*U*^23^
S1	0.0632 (4)	0.0943 (5)	0.0452 (3)	−0.0096 (3)	0.0301 (3)	0.0036 (3)
O1	0.0703 (11)	0.0827 (12)	0.0573 (10)	−0.0268 (9)	0.0324 (9)	−0.0116 (9)
O2	0.0630 (11)	0.0814 (13)	0.0698 (12)	0.0040 (10)	0.0393 (10)	−0.0022 (10)
N1	0.0518 (10)	0.0649 (12)	0.0491 (10)	−0.0074 (9)	0.0269 (9)	−0.0074 (8)
N2	0.0588 (11)	0.0704 (12)	0.0439 (10)	−0.0070 (9)	0.0311 (9)	−0.0025 (8)
N3	0.0611 (12)	0.0780 (14)	0.0419 (9)	−0.0147 (10)	0.0287 (9)	−0.0037 (9)
N4	0.0628 (12)	0.0832 (15)	0.0449 (11)	−0.0118 (11)	0.0299 (10)	−0.0060 (10)
C1	0.0567 (13)	0.0777 (17)	0.0544 (14)	−0.0031 (12)	0.0313 (12)	0.0037 (12)
C2	0.0633 (15)	0.0771 (18)	0.0776 (18)	−0.0037 (14)	0.0436 (15)	0.0078 (14)
C3	0.0841 (19)	0.0761 (18)	0.0785 (19)	0.0119 (15)	0.0603 (17)	0.0164 (14)
C4	0.093 (2)	0.0816 (18)	0.0567 (15)	0.0102 (16)	0.0496 (15)	0.0081 (14)
C5	0.0737 (16)	0.0693 (15)	0.0500 (13)	−0.0021 (13)	0.0381 (12)	−0.0023 (11)
C6	0.0534 (12)	0.0566 (12)	0.0491 (11)	0.0061 (10)	0.0325 (10)	0.0012 (9)
C7	0.0503 (11)	0.0596 (13)	0.0429 (11)	0.0026 (10)	0.0272 (9)	−0.0009 (9)
C8	0.0563 (13)	0.0668 (14)	0.0435 (11)	−0.0018 (11)	0.0263 (10)	−0.0028 (10)
C9	0.0549 (12)	0.0657 (14)	0.0438 (11)	−0.0008 (11)	0.0286 (10)	−0.0044 (10)

### Geometric parameters (Å, °)

**Table:**

S1—C9	1.681 (2)	C2—H2	0.93 (3)
O1—H1A	0.903 (18)	C3—C2	1.378 (4)
O2—H21	0.940 (17)	C3—C4	1.368 (4)
O2—H22	0.907 (18)	C3—H3	0.93 (3)
N1—O1	1.385 (2)	C4—H4	0.92 (3)
N1—C8	1.264 (3)	C5—C4	1.381 (4)
N2—N3	1.362 (3)	C5—H5	0.95 (3)
N2—C7	1.297 (3)	C6—C1	1.389 (3)
N3—H3A	0.923 (17)	C6—C5	1.386 (3)
N4—C9	1.320 (3)	C6—C7	1.483 (3)
N4—H41	0.915 (17)	C7—C8	1.469 (3)
N4—H42	0.930 (17)	C8—H8	0.97 (3)
C1—C2	1.374 (3)	C9—N3	1.355 (3)
C1—H1	0.93 (2)		
			
N1—O1—H1A	104.6 (19)	C3—C4—C5	121.2 (3)
H21—O2—H22	106 (3)	C3—C4—H4	116.6 (19)
C8—N1—O1	112.88 (19)	C5—C4—H4	122.2 (19)
C7—N2—N3	118.58 (19)	C4—C5—C6	120.9 (3)
C9—N3—N2	120.1 (2)	C4—C5—H5	118.5 (18)
C9—N3—H3A	117.9 (16)	C6—C5—H5	120.6 (18)
N2—N3—H3A	122.0 (17)	C1—C6—C7	119.6 (2)
C9—N4—H41	120.3 (17)	C5—C6—C1	117.3 (2)
C9—N4—H42	117.9 (17)	C5—C6—C7	123.0 (2)
H41—N4—H42	121 (2)	N2—C7—C8	125.4 (2)
C2—C1—C6	121.2 (2)	N2—C7—C6	116.00 (19)
C2—C1—H1	118.9 (16)	C8—C7—C6	118.60 (18)
C6—C1—H1	119.7 (16)	N1—C8—C7	122.4 (2)
C1—C2—C3	120.9 (3)	N1—C8—H8	122.8 (16)
C1—C2—H2	118.0 (16)	C7—C8—H8	114.7 (16)
C3—C2—H2	121.1 (16)	N4—C9—N3	117.2 (2)
C4—C3—C2	118.4 (3)	N4—C9—S1	124.28 (17)
C4—C3—H3	120.7 (18)	N3—C9—S1	118.46 (18)
C2—C3—H3	120.8 (18)		
			
O1—N1—C8—C7	177.1 (2)	C1—C6—C5—C4	−0.6 (4)
C7—N2—N3—C9	−175.9 (2)	C7—C6—C5—C4	179.8 (2)
N3—N2—C7—C8	2.8 (3)	C5—C6—C7—N2	177.2 (2)
N3—N2—C7—C6	−179.56 (19)	C1—C6—C7—N2	−2.4 (3)
C6—C1—C2—C3	−0.1 (4)	C5—C6—C7—C8	−5.0 (3)
C4—C3—C2—C1	−0.8 (4)	C1—C6—C7—C8	175.4 (2)
C2—C3—C4—C5	1.0 (4)	N2—C7—C8—N1	−7.6 (4)
C6—C5—C4—C3	−0.3 (4)	C6—C7—C8—N1	174.8 (2)
C5—C6—C1—C2	0.8 (4)	S1—C9—N3—N2	−178.23 (16)
C7—C6—C1—C2	−179.6 (2)	N4—C9—N3—N2	3.0 (3)

### Hydrogen-bond geometry (Å, °)

**Table:**

*D*—H···*A*	*D*—H	H···*A*	*D*···*A*	*D*—H···*A*
O1—H1A···O2^i^	0.90 (3)	1.83 (3)	2.728 (3)	174 (3)
O2—H21···S1	0.94 (3)	2.32 (3)	3.250 (3)	171 (3)
O2—H22···O2^i^	0.91 (3)	1.98 (3)	2.886 (3)	172 (4)
N3—H3A···N1	0.92 (3)	1.91 (2)	2.604 (3)	130 (2)
N4—H41···S1^ii^	0.92 (2)	2.53 (2)	3.434 (2)	169 (3)
N4—H42···N2	0.93 (3)	2.24 (3)	2.643 (3)	105 (2)

Symmetry codes: (i) −*x*+1/2, *y*+1/2, −*z*+3/2; (ii) −*x*, −*y*+1, −*z*+1.

### Table 2 Table 2. Comparison of the bond lengths and angles (Å, °) in the oxime moieties of the title compound,(I), with the corresponding values in the related compounds (II)–(VII)

**Table:**

Bond/Angle	(I)	(II)	(III)	(IV)	(V)	(VI)	(VII)
N1-O1	1.385 (2)	1.403 (2)	1.423 (3)	1.417 (1)	1.429 (4)	1.424 (2)	1.416 (3)
		1.396 (2)	1.396 (3)				1.397 (3)
N1-C8	1.264 (3)	1.281 (2)	1.290 (3)	1.290 (1)	1.241 (6)	1.289 (2)	1.282 (3)
		1.281 (2)	1.282 (3)				1.289 (3)
C7-C8	1.469 (3)	1.477 (3)	1.489 (3)	1.510 (1)	1.551 (7)	1.513 (2)	1.501 (4)
		1.473 (3)					1.502 (4)
C7-C8-N1	122.4 (2)	115.2 (2)	116.6 (2)	114.3 (1)	118.3 (5)	113.2 (1)	114.4 (2)
		115.0 (2)	115.0 (2)				113.4 (2)
C8-N1-O1	112.9 (2)	112.4 (1)	109.4 (2)	110.7 (1)	112.2 (4)	110.6 (1)	110.7 (2)
		112.2 (1)	111.5 (2)				111.1 (2)

Notes: (II): 2,3-dimethylquinoxaline-dimethylglyoxime (1/1) (Hökelek, Batı *et al.*, 2001), (III): 1-(2,6-dimethylphenylamino)propane-1,2-dione dioxime (Hökelek, Zülfikaroğlu & Batı, 2001), (IV): N-hydroxy-2-oxo-2,N'-diphenylacetamidine (Büyükgüngör *et al.*,  2003), (V): N-(3,4-dichlorophenyl)-N'-hydroxy-2-oxo-2-phenylacetamidine (Hökelek *et al.*, 2004), (VI): N-hydroxy-N'-(1-naphthyl)-2-phenylacetamidin-2-one (Hökelek *et al.*,  2004*a*), (VII): N-(3-chloro-4-methylphenyl)-N'-hydroxy-2-oxo-2-phenylacetamidine-2,3- dimethylquinoxaline-dimethyl-glyoxime (1/1) (Hökelek *et al.*, 2004*b*).
